# Single-cell profiling of CD11c+ B cells in atherosclerosis

**DOI:** 10.3389/fimmu.2023.1296668

**Published:** 2024-01-08

**Authors:** Tanyaporn Pattarabanjird, Prasad Srikakulapu, Brett Ransegnola, Melissa A. Marshall, Yanal Ghosheh, Rishab Gulati, Chistopher Durant, Fabrizio Drago, Angela M. Taylor, Klaus Ley, Coleen A. McNamara

**Affiliations:** ^1^ Carter Immunology Center, University of Virginia, Charlottesville, VA, United States; ^2^ Cardiovascular Research Center, University of Virginia, Charlottesville, VA, United States; ^3^ Department of Biomedical Engineering, University of Virginia, Charlottesville, VA, United States; ^4^ Division of Cardiovascular Medicine/Department of Medicine, University of Virginia, Charlottesville, VA, United States; ^5^ La Jolla Institute for Immunology, La Jolla, CA, United States; ^6^ Immunology Center of Georgia, Augusta University, Augusta, GA, United States

**Keywords:** B cells, CITESeq, coronary artery disease, aging, autoimmunity

## Abstract

Circulating CD11c+ B cells, a novel subset of activated B cells, have been linked to autoimmunity and shown to expand with age. Atherosclerosis is an age-associated disease that involves innate and adaptive immune responses to modified self-antigens. Yet, the expression of CD11c on specific B-cell subtypes and its link to atherosclerosis are poorly understood. In this study, we characterized the frequency of CD11c+ B cells in tissues in mice with aging. We observed an age-associated increase in CD11c+ B cells in the spleen and bone marrow of ApoE^−/−^ mice, and this was associated with an increase in aortic plaque. In addition, we also utilized single-cell multi-omics profiling of 60 human subjects undergoing advanced imaging for coronary artery disease (CAD) to subtype CD11c+ B cells and determine their frequency in subjects with high and low severity of CAD. Using unsupervised clustering, we identified four distinct clusters of CD11c+ B cells, which include CD27 and IgD double negative 2 (DN2), age-associated (ABC), CD11c+ unswitched memory (USWM), and activated Naïve (aNav) B cells. We observed an increase in the frequency of both ABC B cells and DN2 B cells in patients with high CAD severity. Pathway analysis further demonstrated augmentation of autophagy, IFNg signaling, and TLR signaling in DN2 cells in high-severity CAD patients. On the other hand, an increase in the negative regulator of BCR signaling through CD72 was found in ABC cells in low-severity CAD patients. Through investigating scRNAseq of atheroma, these DN2 cells were also found to infiltrate human coronary atheroma.

## Introduction

Atherosclerosis is a chronic inflammatory disease, and its incidence increases with age. A wealth of evidence in both preclinical and clinical studies has clearly demonstrated that B cells play important roles in atherosclerosis (1–5). B cells’ effects on atherosclerosis are subtype-dependent and mediated mainly through the production of immunoglobulin and cytokines (6, 7). CD11c+ B cells have been described as proinflammatory B cells through the production of IgG to autoantigens as well as inflammatory cytokines, e.g., IFNg (8, 9). Increases in CD11c+ B cells have been shown to be associated with autoimmune diseases such as systemic lupus erythematosus (SLE) and rheumatoid arthritis (RA), as well as aging (10, 11). Yet, subsets of CD11c+ B cells are poorly defined, and their roles in atherosclerosis are incompletely explored.

Here, we investigated CD11c+ B cells in aged mice. Aged mice with a higher aortic plaque burden were found to have an increase in the frequency of CD11c+ B cells in the blood, spleen, and bone marrow. Through the utilization of single-cell multi-omics sequencing platform (CITESeq), we further subtyped human CD11c+ B cells into age-associated B cells (ABC), double negative 2 (DN2), CD11c+ unswitched memory (USWM), and activated Naïve (aNav). Subjects with severe coronary artery disease (CAD) had a higher frequency of the ABC and DN2 subtype of CD11c+ B cells in circulation compared to those with little to no CAD. Further transcriptomic analysis and flow cytometry characterization identified potential factors that may mediate their impact on atherosclerosis. This study further underscores the importance of CD11c+ B cells in chronic inflammatory diseases like atherosclerosis.

## Materials and methods

### Human subjects

A total of 60 human subjects with varying severity of coronary artery disease (CAD) were enrolled in the study from the Coronary Assessment in Virginia cohort (CAVA) through the Cardiac Catheterization Laboratory at the University of Virginia. Prior to participation, all individuals provided written informed consent without receiving compensation. The study received approval from the Human Institutional Review Board under IRB No. 15328. Peripheral blood samples were collected from these subjects before catheterization.

Subjects were excluded from the study if they exhibited any of the following conditions: acute illness, type 1 diabetes, current acute coronary syndrome (ACS), autoimmune disease, current use of immunosuppressive therapy, previous organ transplantation, anemia, pregnancy, or HIV infection. Following cardiac catheterization, the extent of atherosclerotic disease was quantified using quantitative coronary angiography (QCA) and the Gensini score. Traditional risk factors, including age, BMI, lipid profile, smoking, hypertension, and statin use status, were also assessed through demographic information, physical and physiological measurements, and laboratory values.

### Mice

All animal protocols were approved by the Animal Care and Use Committee at the University of Virginia. Apolipoprotein E-deficient (*ApoE* ^−/−^) mice were purchased from Jackson Laboratory. All mice were fed with a standard chow diet. Mice were euthanized in all experiments with CO_2_ inhalation. Only male mice were used for all experiments.

### Human PBMC isolation

Blood was obtained from both coronary artery disease subjects and collected in BD K2 EDTA vacutainer tubes. This blood was processed at room temperature (RT) within an hour of collection. To eliminate platelet-rich plasma, the whole blood in the vacutainers was subjected to centrifugation at 400×*g* for 10 min at RT. Subsequently, the plasma was cryopreserved at −80°C.

Peripheral blood mononuclear cells (PBMCs) were further separated using Ficoll–Paque density gradient centrifugation, employing Ficoll–Paque PLUS (GE Healthcare Biosciences AB, Chicago, IL) and SepMate-50 (Stemcell Technologies Inc., Cambridge, MA) according to the manufacturer’s instructions. Live cell counts were obtained through Trypan blue staining. These PBMCs were cryopreserved in a freezing solution composed of 90% FBS and 10% DMSO, or they were used fresh. PBMC vials were stored at −80°C initially in a Mr. Frosty (Thermo Fisher, Waltham, MA) for 48 h and were subsequently transferred to liquid nitrogen storage until needed.

### Cell preparation for murine flow cytometry

Peritoneal, spleen, and bone marrow cells were harvested from 50- to 100-week-old ApoE^−/−^ mice and brought into single-cell suspension as previously described (2). Cells were blocked with Fc receptors and then stained for cell surface markers on ice for 20 min. Cells were then washed using 1% BSA-PBS before staining with live-dead aqua (Invitrogen, Waltham, MA) in PBS, resuspended in 1% BSA-PBS, and run on Attune NXT machine. Flow cytometry antibodies for murine cell staining were purchased from Biolegend, San Diego, CA, BD, or Thermo Fisher with the following fluorophore conjugates and clones: B220-APC (RA3-6B2), CD11b-PerCP Cy5.5 (M1/70), CD5-BV421 (53-7.3), CD11c-APC EF780 (N418), IgM-PE (R6-60.2), CD19-PECy7 (1D3), and CD45-PerCP (30F11).

### CITESeq optimization and staining

We obtained PBMCs from 60 CAVA study subjects and performed labeling using the BD Single-Cell Multiplexing Kit (BD Biosciences, San Jose, CA) and CITESeq Ab-Oligos, following the protocol by Vallejo et al. (12). Briefly, PBMCs were thawed at 37°C, washed with complete RPMI-1640 solution, and aliquoted into tubes with 1 million cells each. They were incubated on ice with Fc Block (BD Biosciences, San Jose, CA) in a 1:20 dilution in super bright staining buffer (SB, eBioscience, San Diego, CA), transferred to multiplexing kit tubes (BD Biosciences, San Jose, CA), and incubated at room temperature for 20 min. After three washes and cell viability assessment with DRAQ7 and Calcein AM, the samples were pooled, and 50 unique CITESeq Ab-Oligos (diluted at 2 μL each, table of CITESeq Ab-Oligos were provided in the previous study performed by Pattarabanjird et al. (13)) were added. After incubation on ice for 30–60 min, the cells underwent three more washes and were counted using the BD Rhapsody Scanner.

### CITESeq library preparation

Cell loading at 800–1,000 cells/μL into the primed plate was followed by reverse transcription at 37°C. Exonuclease I was added, and after 30 minutes of incubation, the samples were heated at 80°C for 20 min. The cDNA library was prepared following BD’s protocol as described by Vallejo et al. (12). Quality control and quantification checks were performed using TapeStation and Qubit kits and reagents.

### CITESeq library sequencing

Sequencing was carried out following BD’s recommended depths: Ab-Oligos sequencing (40,000 reads per cell), mRNA sequencing (20,000 reads per cell), and sample tags (600 reads per cell). A total of 60,600 reads per cell were obtained on the NovaSeq. The data were processed using Seven Bridged Genomics pipeline, filtering it into matrices and csv files. The Doublet Finder package in R was employed to eliminate doublets, and cells with <128 antibody molecules sequenced were removed. Antibody sequencing data was CLR normalized and converted to log2 scale, while transcript data were normalized by total UMIs per cell and scaled up to 1,000.

### CITEseq data preprocessing and analysis

B cells within PBMCs were identified through manual gating based on CD19+CD3− antibody sequencing. Antibody expression thresholding was performed on negative cells. UMAP dimensionality reduction and Louvain clustering using 26 B-cell marker antibodies were executed with the Python Scanpy package. Gene expression within B cell subtypes (488 genes) was compared between subjects with low and high coronary artery disease (CAD) using *t*-tests with Bonferroni corrected *p*-values to calculate false discovery rate (FDR) values and fold changes. Differentially expressed genes were defined as those with FDR < 0.05 and Log2FC < −1 or > 1. Volcano plots of differentially expressed genes were visualized with the Python Bioinfokit package. Ingenuity pathway analysis was conducted on all annotated RNA to explore differentially regulated cellular processes and canonical pathways.

### ELISA quantification of IgG and IgM specific to MDA mimotope

The measurement of IgM or IgG specific to MDA mimotope from human plasma was carried out using colorimetric ELISA, as previously detailed (2, 14). In brief, 96-well plates were coated with a concentration of 5 µg/mL of MDA mimotope and incubated overnight at 4°C. Subsequently, the plates were blocked with 1% BSA-PBS before incubation with human serum for a duration of 1.5 h. Detection was achieved using a goat antihuman IgM antibody. 3,3′,5,5′-Tetramethylbenzidine (TMB) was used as a substrate, and horseradish peroxidase (HRP) was used as an enzyme. The ELISA colorimetric assay was read at 450 nm.

### Analysis of atherosclerotic lesions

Heart and aortas were harvested and en face was stained as previously described (15). Plaque area was evaluated using the Image Pro Plus software.

### Statistics

All statistics were performed using GraphPad Prism (version 8.0), Python 3.0, and R 3.6.1. Results were displayed as mean ± SD and a statistical test was described in each figure legend.

## Results

### Murine CD11c+ B cells increase in frequency with age and the degree of atherosclerosis development

**Figure 1:**
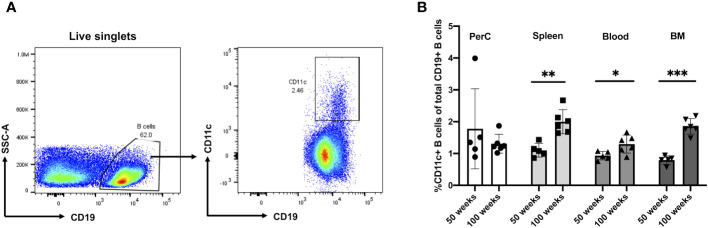
Murine CD11c+ B cell increases in frequency with age in the spleen, blood, and bone marrow. **(A)** Representative flow cytometry gating strategy for CD11c+ B cells in 100-week-old mouse spleen. **(B)** Percentage of CD11c+ B cells of total CD19+ B cells obtained from the peritoneal cavity (PerC), spleen, blood, and bone marrow (BM) of 50- (*n* = 5) and 100-week-old (*n* = 6) ApoE^−/−^ mice. Data in **(B)** were analyzed using the Mann–Whitney Wilcoxon test. Values are mean ± SD. ^*^ *p* < 0.05; ^**^ *p* < 0.01; ^***^ *p* < 0.005.

**Figure 2:**
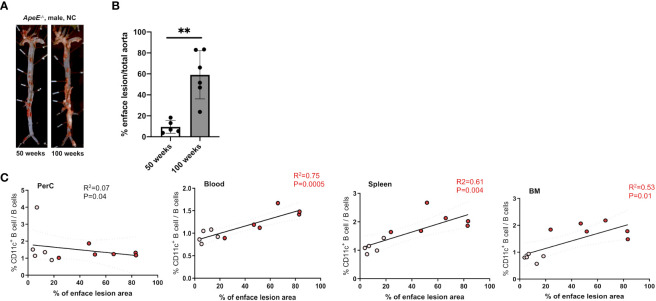
Frequency of CD11c+ B cells in the blood, spleen, and BM correlates with an increase in atherosclerotic area. **(A)** Representative images and enface lesion quantification of aorta obtained from 50-week-old ApoE^−/−^ male mice. **(B)** Percentage of enface lesion of total aorta in 50- (*n* = 5) and 100-week-old (*n* = 6) ApoE^−/−^ male mice. **(C)** Pearson correlations between percentage of CD11c+ B cells of total B cells and percentage of enface lesion area in Perc, blood, spleen, and BM (white dots indicated 50-week-old mice (*n* = 5) and red dot indicated 100-week-old mice (*n* = 6)). The data in **Figure 1A** were analyzed using the Mann–Whitney Wilcoxon test. Values are mean ± SD. ^**^ *p* < 0.01.

To address the frequency and distribution of CD11c+ B cells in relation to aging and atherosclerosis, the frequency of CD11c+ B cells in the peritoneal cavity (PerC), blood, spleen, bone marrow (BM), and aorta was quantified in chow-fed ApoE^−/−^ mice at 50 and 100 weeks of age. CD11c+ B cells were gated as demonstrated in **Figure 1A**. No change in total B-cell frequency was observed between 50- and 100-week-old mice (**Supplementary Figure S1A**). However, a higher percentage of the CD11c+ B cells were found to be higher in the spleen, blood, and BM of 100-week-old mice compared to 50-week-old mice (**Figure 1B**). Harvested aortas were enface stained to quantify atherosclerosis plaque burden (**Figure 2A**). Aged mice at 100 weeks old were found to have a significantly higher atherosclerotic plaque burden compared to 50-week-old mice (**Figure 2B**). Notably, the percentage of B cells that were CD11c+ B cells in blood, spleen, and BM was significantly correlated with atherosclerotic plaque burden (**Figure 2C**), while no correlation was observed between total B-cell frequency and atherosclerotic plaque burden (**Supplementary Figure S1B**). To determine if the variation in atherosclerosis in the 100-week-old mice alone correlated with the percentage of B cells that were CD11c^+^, we analyzed the two age groups separately. A trending positive association between the percentage of B cells that were CD11c^+^ in the blood of the 100-week age group and the level of atherosclerotic burden was found (**Supplementary Figure S2**). No significant correlation was found in other tissue compartments or in the 50-week-old mice, which could be due to being underpowered with a reduced number of mice when separated by age.

### Double negative 2 and age-associated B cells increase in frequency with severe coronary artery disease

**Figure 3:**
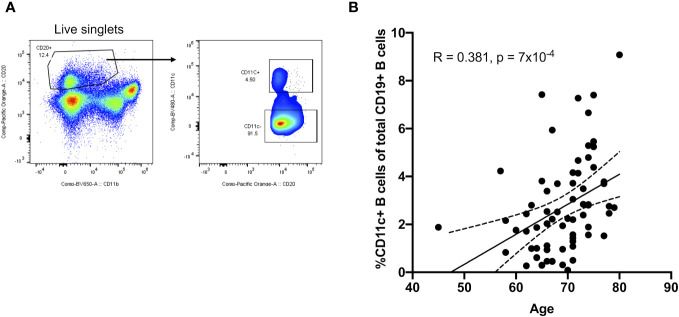
Frequency of circulating CD11c+ B cells increases with age in humans. **(A)** Flow cytometry gating strategy for circulating CD11c+ B cells. **(B)** Pearson correlations between the percentage of circulating CD11c+ B cells in total B cells and age (*n* = 68).

**Figure 4:**
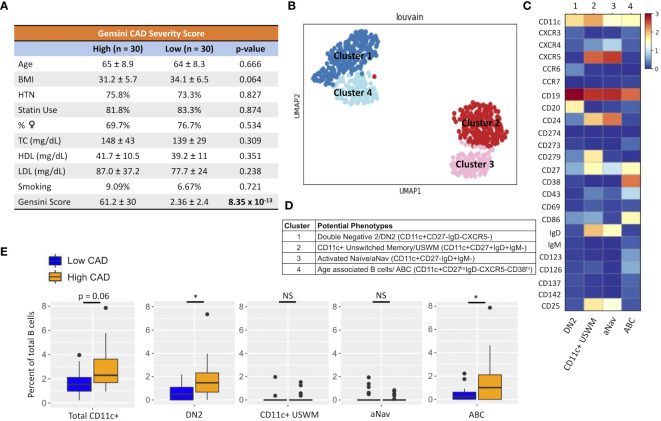
An increase in frequency of double negative 2 (DN2) and age-associated B cells (ABC) was found in patients with high coronary artery disease (CAD) severity. **(A)** Cohort of 60 subjects with high (Gensini score ≥ 32, *n* = 30) and low (Gensini score < 6, *n* = 30) CAD severity with matched age, BMI, hypertension (HTN), %statin use, %female, total cholesterol (TC), HDL cholesterol, LDL cholesterol, and smoking status. **(B)** Representative metacluster UMAO showing four distinct B-cell subsets of circulating CD11c+ B cells by using Louvain clustering. **(C)** Heatmap showing median expression of surface markers from metaclustering. **(D)** Table showing potential phenotypes of each CD11c+ B-cell subset resulting from unsupervised clustering. **(E)** Biaxial plots to compare frequency of each CD11c+ subset as a percentage of total B cells in subjects with high and low CAD severity. Data in **(E)** were analyzed using Mann–Whitney Wilcoxon test. Values are mean ± SD. ^*^ *p* < 0.05.

**Figure 5:**
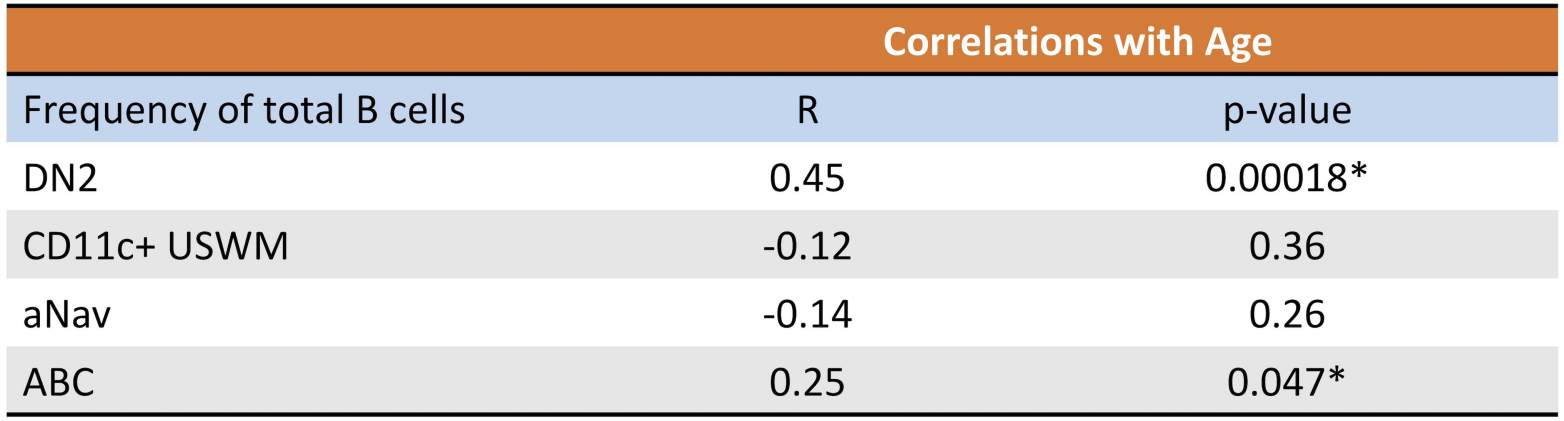
Frequency of circulating DN2 and ABC cells increases with age in humans. Pearson correlations between percentage of circulating DN2, CD11c+ USWM, ABC, and aNav B cells of total B cells and age. *p < 0.05.

Given their potential roles in atherosclerosis demonstrated in mice, we further explore these CD11c+ B cells in humans. Circulating CD11c+ B cells were gated as demonstrated in **Figure 3A** and in a cohort of 60 subjects referred for coronary angiography. We found that the percentage of circulating B cells that were CD11c+ cells significantly increased with age (**Figure 3B**). This 60-subject cohort was selected to test the association of circulating human immune cell subtypes with CAD. The well-established Gensini scoring (16) system was used to divide the cohort into those subjects with low (*n* = 30) and high (*n* = 30) CAD severity. These subjects were otherwise matched for cardiac risk factors, including age, body mass index (BMI), hypertension (HTN), statin use, sex, total cholesterol (TC), HDL, LDL, and smoking status (**Figure 4A**). A 50-antibody and 488-gene single-cell multi-omics sequencing (CITE-Seq) assay was used to subtype the circulating immune cell populations in this cohort. Analysis of other B-cell subtypes, T-cell subtypes, and monocytes has been previously described (13, 17). We utilized this dataset to subtype circulatory CD11c+ B cells in these 60 subjects. Results of Louvain clustering using surface marker antibodies identified four clusters of CD11c+ circulatory B cells (**Figure 4B**). Each cluster was annotated based on expression of known B cell markers represented in the heatmap (**Figures 4C, D**). There were double negative 2 (DN2, CD11c+CD27−IgD−CXCR5−), CD11c+ unswitched memory (CD11c+ USWM, CD11c+CD27+IgD+IgM−), activated naïve (aNav, CD11c+CD27−IgD+), and age-associated B cells (ABC, CD11c+CD27^lo^IgD−CXCR5−). The dot plot showing protein expressions of these hallmark markers, CD11c, CD27, IgD, and CXCR5, to help distinguish these four subtypes of circulating CD11c+ B-cell subtypes was also shown (**Supplementary Figure S3A**). There was a trending increase in total CD11c+ B cells in high CAD severity patients compared to low CAD severity patients (*p* = 0.06). Further subtyping of CD11c+ B cells revealed a significant increase in the DN2 and ABC subtype frequencies in patients with high CAD severity, while no significant difference in frequency was found in CD11c+ USWM and aNav with CAD severity (**Figure 4E**). In addition, the frequencies of human circulating DN2 and ABC were also found to increase with aging (**Figure 5**). As T-bet is a known marker of ABCs but was not in our CITEseq panel, flow cytometry was used to measure T-bet expression across these four CD11c+ B-cell subtypes. Results demonstrate that, compared to total naïve B cells (CD27-IgD+), T-bet is expressed in all CD11c^+^ cells (DN2, CD11c+ USWM, ABC, and aNav cells) (**Supplementary Figure S4**).

**Figure 6:**
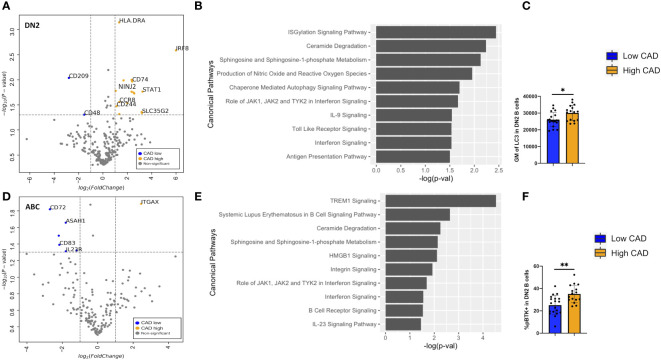
Differentially expressed genes (DEGs) and pathway analyses of DN2 and ABC compared between low and high CAD severity patients. **(A)** Volcano plot of 488 gene array demonstrating DEGs of DN2 when compared between CAD low (blue) and high (orange). **(B)** Significant canonical pathways enriched in DN2 cells. **(C)** Geometric mean (GM) of LC3 performed by flow cytometry in low (n = 19) and high (n = 16) CAD patients. **(D)** Volcano plot of 488 gene array demonstrating DEGs of ABC when compared between CAD low (blue) and high (orange). **(E)** Significant canonical pathways enriched in ABC cells. **(F)** GM of phospho-BTK (p-BTK) performed by flow cytometry in low (n = 19) and high (n = 16) CAD patients. Data in **(C, F)** were analyzed using Mann-Whitney Wilcoxon test. Values are mean ± s.d.. *p < 0.05, **p < 0.001.

Differentially expressed gene (DEG) analyses of the 488 genes were performed on DN2 and ABCs. For DN2, there were 24 DEGs (FDR < 0.05 and log_2_FC > or < 1) compared between low and high CAD severity patients (**Supplementary Table S1**). The annotated volcano plot (**Figure 6A**) highlighted an enrichment of interferon regulatory factor 8 (IRF8), CD74, Ninjurin 2 (NINJ2), and STAT1 in DN2 cells of patients with severe CAD. Ingenuity pathway analysis of DEG in DN2 cells identified processes such as autophagy signaling, toll-like receptor (TLR), and interferon-gamma (IFN-g) signaling as significant pathways (**Figure 6B**). In addition, we also found higher expression of the LC3 protein, measured using flow cytometry (**Figure 6C**), in DN2 cells from subjects with high-severity CAD. LC3 is a protein mediating autophagosome formation. An analysis of DEG in ABCs revealed nine DEGs (FDR < 0.05 and log_2_FC > or < 1) when compared between low and high CAD severity patients (**Supplementary Table S2**). The volcano plot highlights CD72, a BCR co-receptor downregulating BCR signaling, to be increased in expression in low CAD severity patients, and ITGAX or CD11c, a B-cell activation marker, to be increased in high CAD severity patients (**Figure 6D**). Pathway analysis of DEGs in ABCs showed canonical pathways such as HMGB1 and BCR signaling to be significantly different (**Figure 6E**). Further evaluation utilizing flow cytometry, revealed a greater percentage of ABC cells that were phospho-BTK+ (p-BTK), a downstream signaling of BCR activation, in ABCs from subjects with high CAD severity (**Figure 6F**).

### DN2 infiltrates coronary atheroma and associates with high atherogenic IgG to MDA plasma level

**Figure 7:**
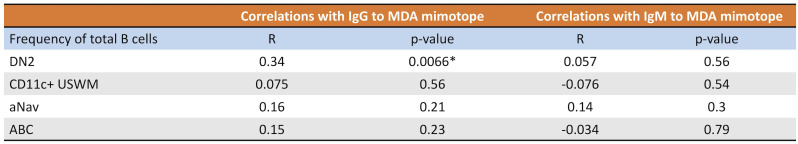
DN2’s frequency directly correlates with plasma IgG and MDA levels. Pearson correlations of DN2, CD11c+ USWM, aNav, and ABC’s frequency with plasma IgG to MDA or plasma IgM to MDA levels. *p < 0.05.

CD11c+ B cells are known to mediate inflammation via autoantibody production (18). Malondialdehyde low-density lipoprotein (MDA-LDL) is a known oxidation-specific epitope deposited in atherosclerotic plaque (14). A wealth of evidence has shown that IgM to MDA-LDL is atheroprotective, while IgG to MDA-LDL is likely atherogenic (19–22). Since MDA-LDL is a high variability antigen with limited reproducibility, MDA-mimotope, a 12-amino acid linear peptide (HSWTNSWMATFL), was engineered (14) to mimic the MDA epitope and has been widely used for both *in vitro* and *in vivo* assays (1, 23–25). Therefore, we further evaluated the relationship between subtypes of CD11c+ B cells and plasma levels of IgG and IgM specific to MDA-mimotope. The frequency of DN2 was found to be directly associated with the plasma level of IgG to MDA-mimotope, while other CD11c+ B-cell subtypes demonstrated no significant correlations with IgG nor IgM to MDA-mimotope (**Figure 7**).

**Figure 8:**
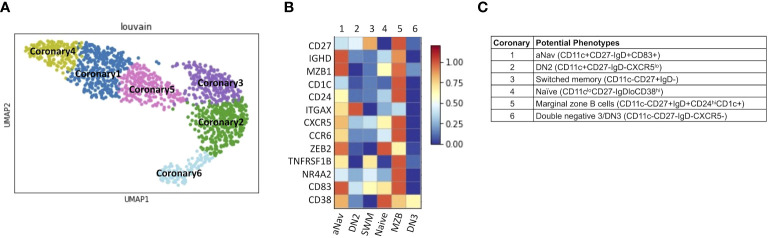
DN2 and aNav B cells were found in human coronary atheroma. **(A)** Representative metaclustering UMAP showing six distinct B-cell subsets obtained from scRNAseq of fresh human coronary atheroma (*n* = 3). **(B)** Heatmap showing median expression of surface markers from metaclustering. **(C)** Table showing potential phenotypes of each B-cell subset resulting from unsupervised clustering. ScRNAseq data of human fresh coronary atheroma was obtained from publicly available data at GEO project number GSE179159 performed by Jaiswal et al.

Lastly, we explored B-cell subtypes in fresh coronary atheroma. scRNAseq performed on sorted CD45+ from fresh coronary atheroma (*n* = 3) and deposited by Jaiswal et al. at GSE179159 was used to re-cluster MS4A1+ B cells. Louvain clustering revealed six subpopulations of B cells, naming coronary 1–6 on UMAP (**Figure 8A**). Mean expressions of known B-cell markers were demonstrated by the heatmap (**Figure 8B**). The six subtypes of B cells in coronary atheroma annotated by these known B cell markers included aNav (CD11c+CD27−IgD+CD83+), DN2 (CD11c+CD27−IgD−CXCR5^lo^), switched memory (CD11c−CD27+IgD−), naïve (CD11c^lo^CD27−IgD^lo^CD38^hi^), marginal zone B cells (CD11c−CD27+IgD+CD24^hi^CD1c+), and double negative 3/DN3 (CD11c−CD27−IgD−CXCR5−) (**Figure 8C**). A dot plot of transcript expression of ITGAX/CD11c, CXCR5, CD27, and IGHD/IgD is also shown in **Supplementary Figure S3B**. These results altogether suggested that DN2, which was found to have a higher frequency in patients with high atherogenic IgG-to-MDA levels, also infiltrates coronary atheroma. DEG analysis of coronary atheroma’s DN2 cells compared to the rest of the B-cell subtypes revealed 11 DEGs with higher expression of FCRL5, SOX5, and IRF4 consistent with previously published data by Jenk et al. (11) (**Supplementary Table S3**).

## Discussion

Atherosclerosis is a chronic inflammatory disease driven by aging. CD11c+ B cells increase with age, autoimmune disease, and chronic infection (8, 26, 27). Therefore, this study aimed to further explore the roles of these CD11c+ B cells in atherosclerosis by utilizing both an ApoE^−/−^ aged mouse model as well as CITESeq and scRNAseq to study these B-cell subpopulations in humans.

Through characterizing B cells obtained from 50- and 100-week-old ApoE^−/−^ mice, CD11c+ B cells were found to reside in PerC, blood, spleen, and bone marrow. More importantly, the frequency of these cells in blood, spleen, and bone marrow was found to be higher in 100-week-old mice compared to 50-week-old mice (**Figure 1B**). This result was in agreement with the recent work by Smit et al. that demonstrated an increase in CD11b+CD11c+Tbet+ cells in atherosclerotic aorta in 22-month-old Ldlr^−/−^ mice (28). In addition, these CD11c+ B cells from combining 50- and 100-week-old mice obtained from spleen, blood, and bone marrow were also found to significantly correlate with high aortic plaque burden. However, only circulating CD11c+ B cells were found to be trending significantly within the 100-week-old group, consistent with our human data (**Supplementary Figure S2**). These correlation studies underscore the importance of future studies to prove the causal effect of these CD11c+ B cells on atherosclerotic plaque formation.

To further investigate CD11c+ B cells in human atherosclerosis, CITESeq of 60 CAD patients with low and high CAD severity was used to further subtype and characterize CD11c+ B cells. Our study was the first to demonstrate that DN2 B cells, which were known to expand in autoimmune diseases such as RA and SLE (10, 11), also increase with aging and with high CAD severity. These DN2 cells could potentially contribute to an increase in atherosclerotic burden through the production of atherogenic IgG to MDA, as the frequency of DN2 cells was significantly correlated with plasma IgG to MDA (**Figure 6**). This result is consistent with a previous study by Frasca et al. that showed a significant decrease in IgG-to-MDA production once DN cells were removed (18). IgG-to-MDA production by DN2 is likely through T-cell help. Further validation of the associative data using *in vivo* study is needed to show the functional production of IgG to MDA by DN2 cells.

Within the DN2 population, transcriptome analysis also revealed high expression of IRF8, STAT1, CD74, and NINJ2 in patients with high CAD severity (**Figure 6A**). CD74, or MHCII invariant chain, has an enigmatic role in B-cell survival, proliferation, and antigen presentation (29) while also enabling TLR4 signaling (30, 31). STAT1 has been known as a downstream TLR signaling molecule, and NINJ2, or ninjurin2, has been recognized as a TLR4 regulator in vascular endothelial cells, leading to inflammation in atherosclerosis (32). Altogether, these enriched transcriptomes suggested an increase in TLR signaling in high-CAD-severity patients, which aligned with the result from pathway analysis (**Figure 6B**). This discovery is consistent with prior studies showing the importance of TLR signaling in the progression of atherosclerosis (33) and TLR7/9 signaling as a potential major contributor to the development of CD11c+ B cells (34, 35). On the other hand, IRF8, another transcriptome enriched in severe atherosclerosis, was previously shown to be an upstream mediator of LC3, leading to autophagosome formation (36). Consistently, intracellular staining of LC3 demonstrated an increase in LC3 expression in patients with severe atherosclerotic burden (**Figure 6C**). Autophagy has been suggested as a potential cellular mechanism enabling cells to survive longer and proliferate. It is possible that autophagy in DN2 facilitates longer B-cell survival, leading to higher production of atherogenic IgG to MDA, resulting in an increased atherosclerotic burden. Further study is needed to validate this hypothesis.

In the case of the ABC population, transcriptome analysis indicated higher expression of ITGAX or CD11c in high-CAD-severity patients, while higher expression of CD72, a negative regulator of BCR signaling, was found in CAD patients with low severity (**Figure 6D**). These DEGs and pathway analyses suggested that BCR signaling may be enriched in patients with severe atherosclerosis. Intracellular staining of p-BTK showing higher p-BTK in ABC of high-CAD-severity patients (**Figure 6E**) also supports his hypothesis. Although ABC frequency did not show any significant associations with IgG or IgM to MDA antigen, it is possible that these ABC might produce other immunoglobulins to other atherosclerosis-related antigens or other self-neoantigens. A broad IgG and IgM array is required to further evaluate this potential mechanism.

Since MDA is a known antigen deposited in atherosclerotic plaque (14) and DN2 might potentially produce IgG to MDA (**Figure 7**), we investigated publicly available scRNAseq data obtained from fresh atheroma. A previous study by Smit et al. has shown that CD11c+ B cells infiltrate carotid atheroma (28). Our study suggested that DN2 but not ABC infiltrated the coronary atheroma. Previous studies identified arterial tertiary lymphoid organs (ATLO) in the aorta and fat associated lymphoid clusters (FALCS) in the perivascular adipose tissue (PVAT) as a predominant sources harboring most artery-associated B cells, while fewer B cells infiltrate into plaque regions (37, 38). Therefore, it is possible that subtypes of B cells found in coronary atheroma might not represent the majority of B cells orchestrating atherosclerotic plaque. A future study comparing B cells in human coronary atheroma and the PVAT region is required to evaluate this possibility.

Protein expression of CXCR5 in circulating DN2 was found to be different from CXCR5 transcript expression in DN2 obtained from coronary atheroma (**Figures 4C**, **8B**; **Supplementary Figure S3**). These differences in expressions might be explained by the fact that only a proportion of CXCR5 transcript is translated into the CXCR5 protein. Another potential reason is that CXCR5 expression might be upregulated in coronary atheroma compared to circulatory B cells, given that all B-cell subtypes in coronary atheroma were observed to express CXCR5 transcript. However, further study showing both transcript and protein expressions of CXCR5 in blood and atheroma is required to validate this hypothesis.

In summary, our study utilized single-cell profiling as well as an aged mouse model to further characterize CD11c+ B cells in the context of atherosclerosis. The aged mouse model showed an expansion of CD11c+ B cells in the spleen, blood, and bone marrow, which was highly associated with atherosclerotic plaque burden. Additionally, CITESeq also identified DN2 and ABC subtypes of CD11c+ B cells to be associated with severe atherosclerosis in humans. Transcriptome and pathway analysis unveiled that TLR signaling and autophagy might contribute to DN2 cell proliferation and atherogenic IgG to MDA antibody production. ScRNAseq analysis also suggested that these DN2 cells also infiltrate the coronary plaque region. These findings underscore the potential significance of DN2 cells in modulating chronic inflammation in atherosclerosis.

## Data availability statement

The original contributions presented in the study are publicly available. This data can be found here: National Center for Biotechnology Information (NCBI) Gene Expression Omnibus (GEO), https://www.ncbi.nlm.nih.gov/geo/, GSE190570 and GSE179159.

## Ethics statement

The studies involving humans were approved by Human Institutional Review Board. The studies were conducted in accordance with the local legislation and institutional requirements. The participants provided their written informed consent to participate in this study. The animal study was approved by Animal Care and Use Committee at the University of Virginia. The study was conducted in accordance with the local legislation and institutional requirements.

## Author contributions

TP: Conceptualization, Data curation, Formal analysis, Investigation, Methodology, Validation, Visualization, Writing – original draft, Writing – review & editing. PS: Conceptualization, Formal analysis, Investigation, Validation, Visualization, Writing – review & editing. BR: Methodology, Validation, Writing – review & editing. MM: Methodology, Writing – review & editing. YG: Formal analysis, Writing – review & editing. RG: Formal analysis, Writing – review & editing. CD: Methodology, Writing – review & editing. FD: Formal analysis, Writing – review & editing. AT: Resources, Writing – review & editing. KL: Funding acquisition, Resources, Writing – review & editing. CM: Conceptualization, Funding acquisition, Resources, Writing – review & editing.
