# Supplementary material for: Single-cell profiling of CD11c+ B cells in atherosclerosis
Source: Front Immunol. 2024 Jan 8;14:1296668. doi: 10.3389/fimmu.2023.1296668 (PMC10800418; doi:10.3389/fimmu.2023.1296668)
Supplement: Supplementary file 1 [file DataSheet_1.pdf]

Supplementary table 1: Differentially expressed genes from DN2 B cells compared between patients with low and high CAD severity

Gene Names	p-val	log2FC
HLA.DRA	0.00072047	1.33716249
IRF8	0.00259445	6.0025725
HMMR	0.00639085	0.44093532
CD209	0.00919843	-2.793172
NINJ2	0.01006105	2.35711422
S100A10	0.01030268	1.69702371
CD74	0.01081566	2.40555662
MXD1	0.01663882	1.0479823
STAT1	0.01721314	3.24962371
SNCA	0.01733457	2.35726117
GNG11	0.01808265	2.52321843
SDCBP	0.01876894	2.57549483
CD79B	0.0219355	0.44305717
CD79A	0.02636671	0.19499874
CCR8	0.02772215	1.35537494
ASAH1	0.02926546	2.01467081
HLA.DPA1	0.03269616	0.37003132
KLRB1	0.03286468	0.62984825
CD244	0.03370871	1.11656095
HLA.DQB1	0.03581049	0.29673629
GIMAP5	0.04405295	3.18725062
SLC35G2	0.04588601	3.20708829
FAS	0.04844288	1.34569013
CD48	0.04976103	-1.5336218

Supplementary table 2: Differentially expressed genes from DN2 B cells compared between patients with low and high CAD severity

Gene Names	pval	log2FC
ITGAX	0.01301334	2.4559812
HLA.A	0.01333807	-0.4116038
CD72	0.01516943	-2.6985889
ASAH1	0.02203375	-1.8035154
HLA.B	0.03155565	-0.2641844
NR4A1	0.03162976	-2.1984013
CD83	0.04076289	-2.1662687
TRAC	0.04787909	-1.1889541
IL23R	0.04879276	-1.798395

Supplementary table 3: Differentially expressed genes from DN2 B cells compared to the rest of B cell subtypes

Gene Names	log_2foldchanges	p-values
HLA-DRB5	1.5496678	1.44E-12
SOX5	1.5526713	3.80E-08
GZMA	1.6455833	7.36E-05
FCRL5	1.63959	0.01861441
XCL1	2.3278227	0.02025352
NAPRT	1.3834691	0.02880386
ITGAX	1.6177727	0.05314026
LMNA	0.80564034	0.06857436
IRF4	23.180809	0.08326294
LINC01358	2.7341955	0.0962317
CA2	2.7341955	0.0962317
