# Supplementary figures and images for: Single-cell profiling of CD11c+ B cells in atherosclerosis
Source: Front Immunol. 2024 Jan 8;14:1296668. doi: 10.3389/fimmu.2023.1296668 (PMC10800418; doi:10.3389/fimmu.2023.1296668)

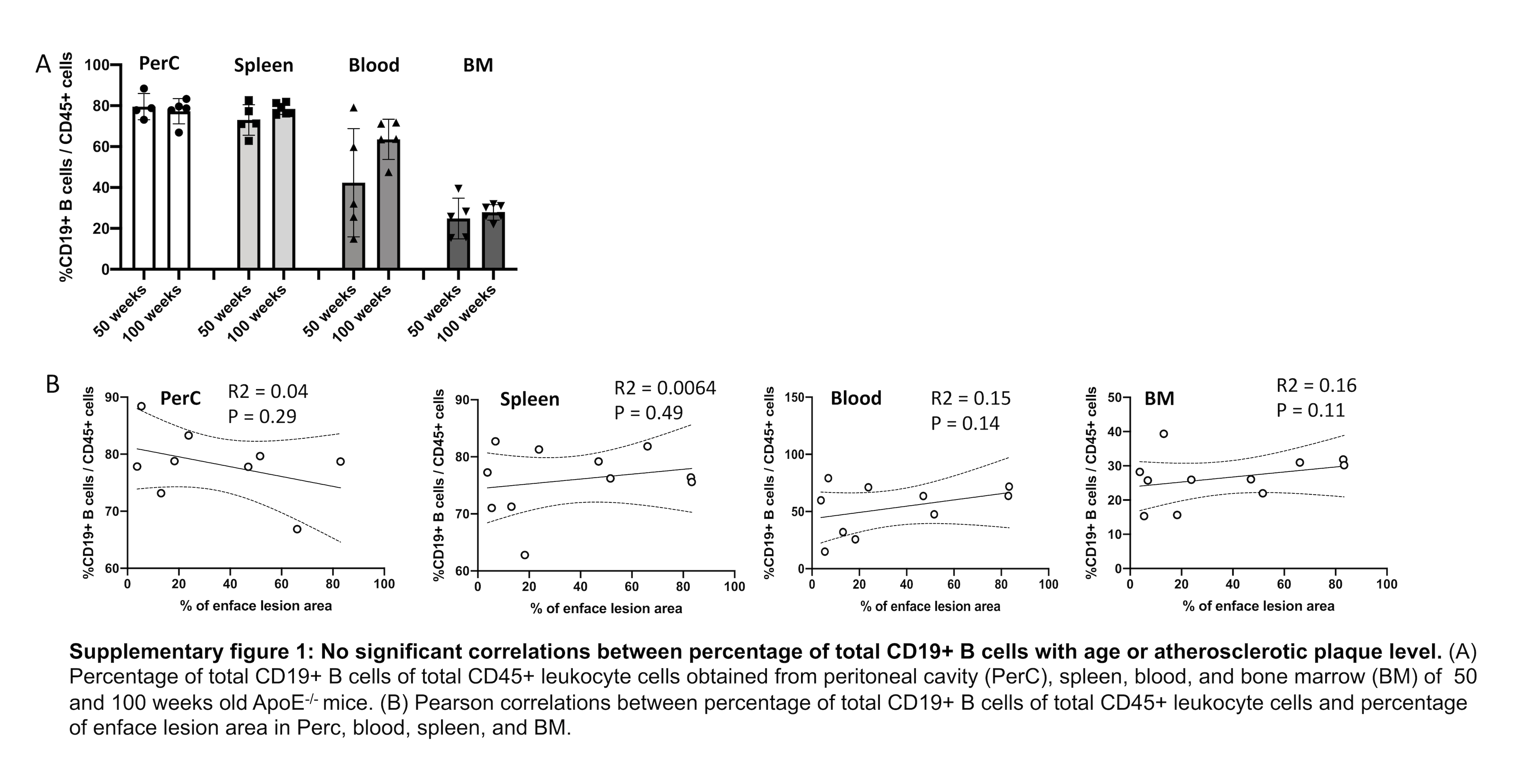

Supplement: Supplementary file 2 [file Image_1.jpeg]

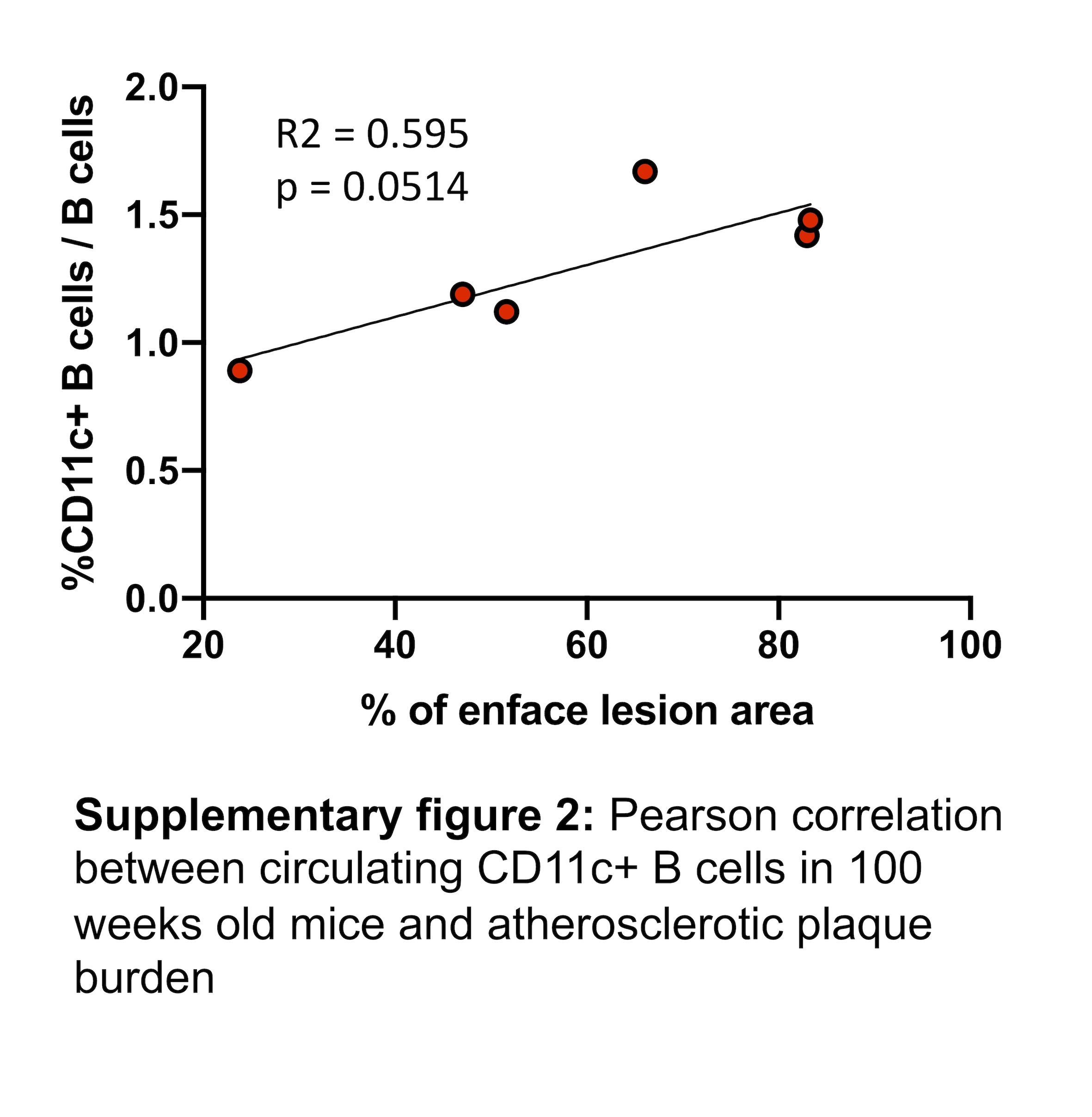

Supplement: Supplementary file 3 [file Image_2.jpeg]

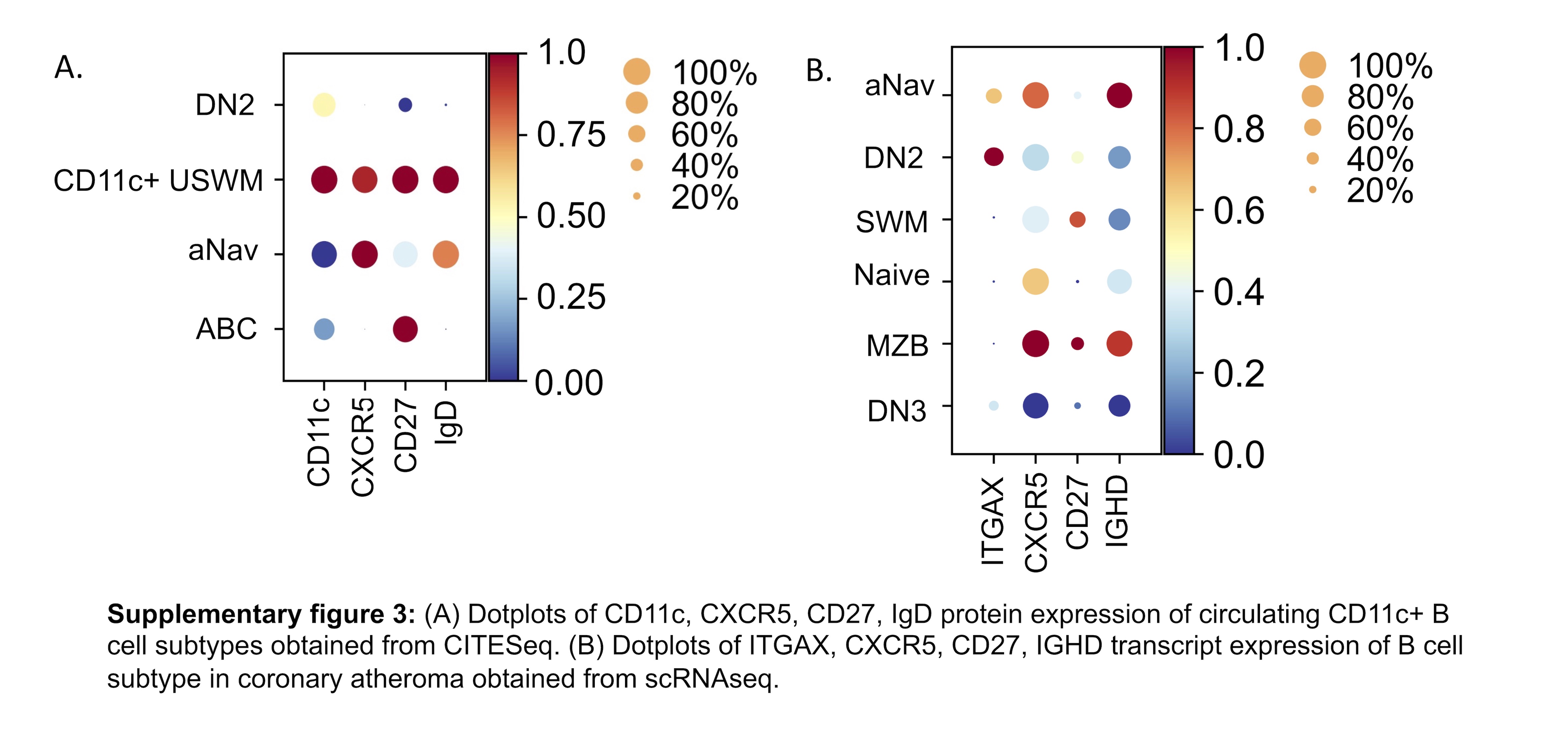

Supplement: Supplementary file 4 [file Image_3.jpeg]

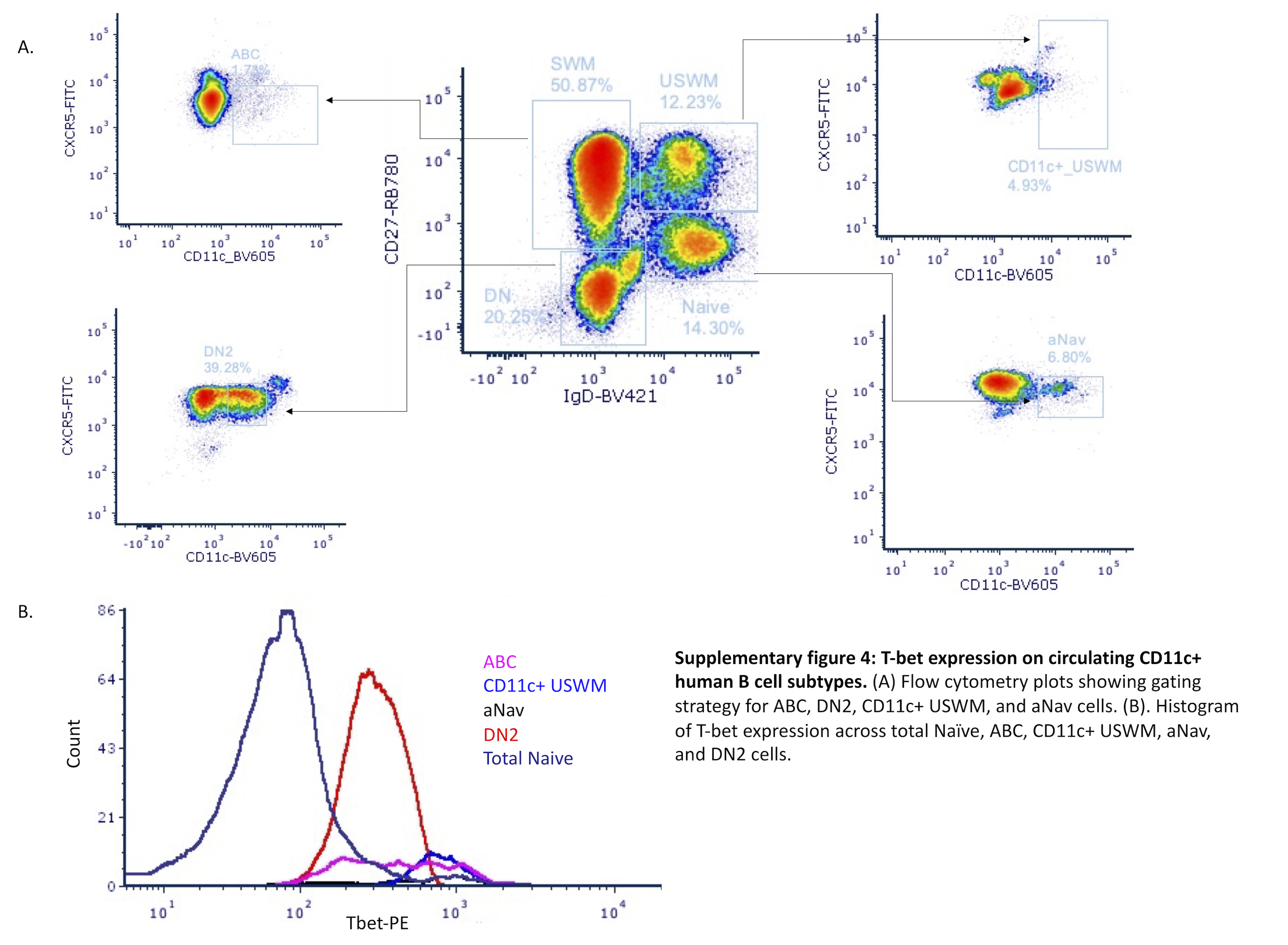

Supplement: Supplementary file 5 [file Image_4.jpeg]
